# Consecutive Three-Component Synthesis of 3-(Hetero)Aryl-1*H*-pyrazoles with Propynal Diethylacetal as a Three-Carbon Building Block

**DOI:** 10.3390/molecules16119340

**Published:** 2011-11-07

**Authors:** Lucilla Levi, Christina Boersch, Charlotte F. Gers, Eugen Merkul, Thomas J. J. Müller

**Affiliations:** Institut für Organische Chemie und Makromolekulare Chemie, Heinrich-Heine-Universität Düsseldorf, Universitätsstraße 1, D-40225 Düsseldorf, Germany

**Keywords:** alkyne, cyclocondensation, multi-component reaction, pyrazole, Sonogashira coupling

## Abstract

A novel consecutive three-component synthesis of 3-(hetero)aryl-1*H*-pyrazoles via room temperature Sonogashira arylation of propynal diethylacetal used as a propargyl aldehyde synthetic equivalent has been disclosed. The final acetal cleavage-cyclocondensation with hydrazine hydrochloride at 80 °C rapidly furnishes the title compounds in a one-pot fashion.

## 1. Introduction

Pyrazoles, *i.e.*, diazoles with two adjacent nitrogen atoms, display a rich chemistry and are used in numerous applications [1,2,3]. Their broad spectrum of biological activities, such as anti-hyperglycemic, analgesic, anti-inflammatory, antipyretic, anti-bacterial, and sedative-hypnotic properties, has attracted considerable interest in medicinal chemistry [4,5,6]. In addition, several 3,5-diaryl substituted pyrazoles also reversibly inhibit monoamine oxidases A and B [7]. In crop protection, 1,4-dialkyl-3,5-diphenylpyrazoles are known as potent herbicides [8]. Furthermore, pyrazoles are pluripotent as ligands in coordination chemistry [9,10,11,12], building blocks in heterocyclic synthesis [13,14], and units in supramolecular entities [15,16,17]. In addition, they have received attention as optical brighteners [18] and UV stabilizers [19] as well as photoinduced electron transfer systems [20,21].

Since multi-component reactions (MCRs) are highly efficient and address the fundamental principles of chemo-, regio-, and stereoselectivity, they are receiving an increasing interest in academia and industry [22,23,24,25,26,27,28,29,30,31,32,33,34,35]. With respect to economical and ecological considerations conceptually novel synthetic approaches [36,37,38,39] are directed towards the improvement of processes [40,41], while simultaneously reducing the input of work and the generation of waste. Therefore, MCRs are not only elegant, but also beneficial for the environment [39].

In the past years, we have developed and elaborated the concept of MCR syntheses of multiple classes of heterocycles via Sonogashira coupling of alkynes and (hetero)aroyl chlorides with alkynyl ketones, followed by Michael addition-cyclocondensation, eventually in a one-pot fashion [42,43,44,45]. Alkynyl ketones and aldehydes are ideal three-carbon building blocks as synthetic equivalents of β-dicarbonyl compounds. However, their electrophilicity is enhanced and the inherent regioselectivity is imposed in the Michael-type nucleophilic additions. Within this conceptual framework we have devised regioselective three-component [46] and four-component syntheses [47] of highly luminescent tri- and tetrasubstituted pyrazoles. Here, we communicate a straightforward consecutive three-component synthesis of 3-(hetero)aryl-1*H*-pyrazoles, *i.e*., monosubstituted pyrazole derivatives, via the concatenation of a Sonogashira alkynylation of (hetero)aryl iodides with the commercially available propynal diethylacetal, an *in situ* acetal cleavage, and a final cyclocondensation with hydrazine hydrochloride.

## 2. Results and Discussion

**Scheme 1:**
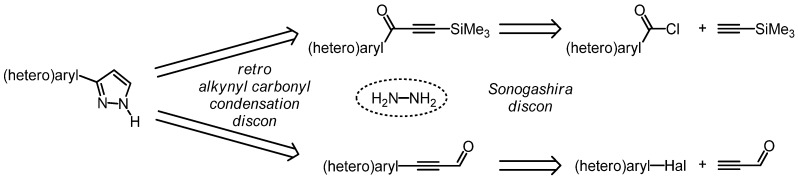
Retrosynthetic conception of three-component pyrazole syntheses by virtue of alkynyl carbonyl condensation.

Monosubstituted 3-(hetero)aryl pyrazoles can be retrosynthetically traced back either to (hetero)aroyl chlorides and TMS-acetylene via 3-trimethylsilylalk-2-yn-1-ones or to (hetero)aryl halides and propargyl aldehyde (Scheme 1).

Both disconnection approaches proceed via a retro alkynyl carbonyl condensation, thus leading to hydrazine and either to an alkynyl ketone [46,47,48] or a propargyl aldehyde [49,50,51,52,53,54]. While the former disconnection establishes the three-carbon skeleton by virtue of Sonogashira acylation [46,55,56], the latter analysis takes advantage of the ligation of the aryl substituent to a propargyl aldehyde synthon via Sonogashira arylation. Since the direct alkynylation of propargyl aldehyde has not been reported, presumably due to its pronounced electrophilicity, propynal diethylacetal apparently represents a suitable synthetic equivalent [57,58,59]. Although the most recent report on the coupling of aryl halides with propynal diethylacetal taking advantage of a tetradentate phosphane ligand based upon the cyclopentane scaffold affords high yields and requires low catalyst loadings, the requisite high reaction temperatures are less favorable for thermally sensitive functionalities [57].

**Scheme 2:**
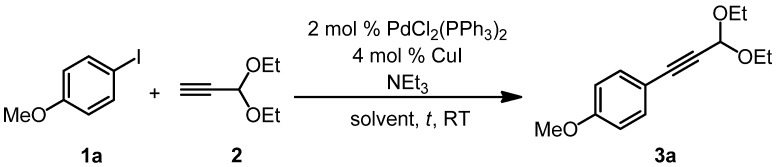
Optimization of the Sonogashira coupling of *p*-iodoanisole (**1a**) and propynal diethylacetal (**2**) to furnish 1-(*p*-anisyl)-3,3-diethoxyprop-1-yne (**3a**).

Therefore, we first set out to optimize the reaction conditions for the Sonogashira coupling step. The transformation of *p*-iodoanisole (**1a**) and propynal diethylacetal (**2)** into 1-(*p*-anisyl)-3,3-diethoxyprop-1-yne (**3a**) under standard Sonogashira conditions at room temperature was chosen as a model reaction (Scheme 2). The amount of triethylamine, the solvent, and the reaction time were modified (Table 1).

**Table 1:** Optimization study for the synthesis of 1-(*p*-anisyl)-3,3-diethoxyprop-1-yne (**3a**).

Entry ^1^	Alkyne 2	NEt_3_	Solvent	Reaction time	Yield of 3a ^2^
1	1.0 equiv.	2.0 equivs.	THF	2 h	64%
2	1.0 equiv.	2.0 equivs.	1,4-dioxane	2 h	80%
3	1.1 equivs.	1.1 equivs.	1,4-dioxane	2 h	64%
**4 ^3^**	**1.1 equivs.**	**2.0 equivs.**	**1,4-dioxane**	**2 h**	**89%**
5	1.1 equivs.	2.0 equivs.	1,4-dioxane	1 h	87%
6	1.1 equivs.	2.0 equivs.	1,4-dioxane	3 h	91%

^1^ The reactions were carried out on a 2.0 mmol scale (*c*(**1a**) = 0.4 M); ^2^ All yields were determined after isolation and purification on silica gel; ^3^ This reaction was additionally carried out on a 5.0 mmol scale [*c*(**1a**) = 0.4 M] yielding 81% of **3a**.

1,4-Dioxane is apparently a better solvent for the coupling than THF (Table 1, entries 1 and 2), a small excess of alkyne **2** furnishes a higher yield (Table 1, entry 3), and two equivalents of triethylamine are more favorable for complete conversion (Table 1, entries 4–6), which is reached after 2 h (Table 1, entry 4). Therefore, the parameters of entry 4 were considered to be optimal for the first step of the sequence (*vide infra*).

**Scheme 3:**
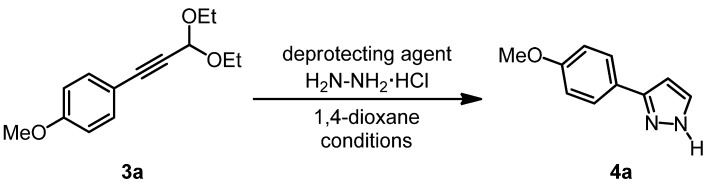
Optimization of the acetal cleavage-cyclocondensation of 1-(*p*-anisyl)-3,3-diethoxyprop-1-yne (**3a**) to furnish 3-(*p*-anisyl)-1*H*-pyrazole (**4a**).

Due to the sensitivity of the deprotected 3-aryl propargyl aldehydes towards oligo- and polymerization in the presence of nucleophiles we then addressed the sequential acetal cleavage-cyclocondensation of 1-(*p*-anisyl)-3,3-diethoxyprop-1-yne (**3a**) in the presence of hydrazine hydrochloride [60,61,62] giving rise to the formation of 3-(*p*-anisyl)-1*H*-pyrazole (**4a**) (Scheme 3, Table 2).

The optimal temperature of the sequential acetal cleavage-cyclocondensation is 80 °C (Table 2, entries 1–4), as preliminary experiments showed that lower temperatures yield poorer results and higher temperatures lead to no further improvement. Conductive heating (Table 2, entries 2–4) turns out to be superior to dielectric heating in the microwave oven (Table 2, entry 1).

**Table 2:** Optimization study for the synthesis of 3-(*p*-anisyl)-1*H*-pyrazole (**4a**).

Entry	Deprotecting agent ^1^	H_2_N-NH_2_·HCl	Reaction temperature	Reaction time	Yield of 4a ^2^
1 ^3^	H_2_O	2.0 equivs.	80 °C (MW)	15 min	53%
**2 ^4^**	**H_2_O**	**2.0 equivs.**	**80 °C ^5^**	**15 min**	**65%**
3 ^3^	H_2_O	2.0 equivs.	80 °C ^5^	1 h	62%
4 ^4^	H_2_O	1.0 equiv.	80 °C ^5^	15 min	22%
5 ^3^	HCl_aq_ (1 *N*)	2.0 equivs.	130 °C ^5^	10 min	55%

^1^ 2.5 mL/mmol were added; ^2^ All yields were determined after isolation and purification on silica gel; ^3^ The reactions were carried out on a 0.5 mmol scale [*c*(**3a**) = 0.4 M]; ^4^ The reactions were carried out on a 1.0 mmol scale [*c*(**3a**) = 0.4 M]; ^5^ Preheated oil bath.

**Scheme 4:**
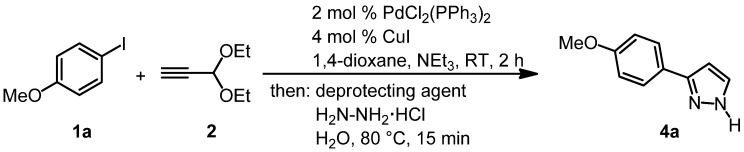
Optimization of the consecutive three-component Sonogashira coupling-acetal cleavage-cyclocondensation synthesis of 3-(*p*-anisyl)-1*H*-pyrazole (**4a**).

While the complete conversion is already achieved within 15 min (Table 2, entries 2 and 3), the amount of hydrazine hydrochloride is crucial for the success of the sequence (Table 2, entries 2 and 4). Strongly Brønsted acidic conditions (Table 2, entry 5) are not required and the combination of water and hydrazine hydrochloride is sufficient to trigger the acetal cleavage and the Michael addition followed by cyclocondensation. Thereafter, the combination of Sonogashira coupling and sequential acetal cleavage-cyclocondensation was performed with the established model system (Scheme 4, Table 3).

**Table 3:** Optimization study for the one-pot synthesis of 3-(*p*-anisyl)-1*H*-pyrazole (**4a**).

Entry	Deprotecting agent	Yield of 4a ^1^
1		not isolated
2	1.0 equiv. of HCl_aq_ (1 *N*)	50%
3	1.0 equiv. of CH_3_COOH	25%
**4**	**1.0 equiv. of PTSA·H_2_O**	**56%**
5	2.0 equivs. of PTSA·H_2_O	55%

^1^ All yields were determined after isolation and purification on silica gel.

Although the acetal cleavage-cyclocondensation conditions only require a moderate acidity (*vide supra*), the excess triethylamine from the coupling step has to be buffered (Table 3, compare entry 1 with entries 2–5), which is most efficiently achieved by addition of a stoichiometric amount of *p*-toluenesulfonic acid (PTSA) (Table 3, entry 4).

**Scheme 5:**
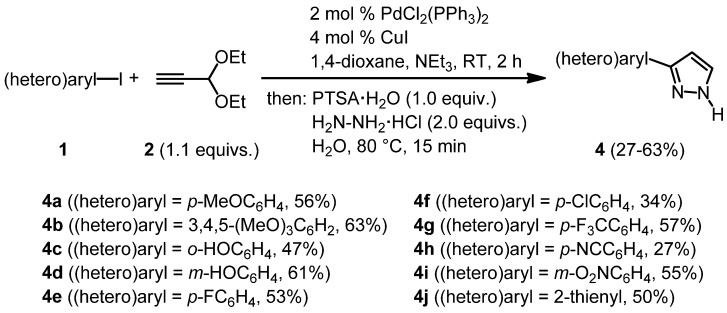
Consecutive three-component Sonogashira coupling-acetal cleavage-cyclocondensation synthesis of 3-(hetero)aryl-1*H*-pyrazoles **4**.

With these optimized conditions for the whole sequence in hand, the stage was set for testing the range of applicable (hetero)aryl iodides **1** in this novel consecutive three-component synthesis of 3-(hetero)aryl-1*H*-pyrazoles **4**. After coupling of (hetero)aryl iodides **1** with propynal diethylacetal (**2**) at room temperature for two hours the subsequent addition of PTSA·H_2_O, hydrazine hydrochloride, and water, heating to 80 °C for 15 min gave rise to the isolation of 3-(hetero)aryl-1*H*-pyrazoles **4** in moderate to good yields (Scheme 5).

This first study on the methodological scope of this novel three-component pyrazole synthesis shows that electron-rich as well as electron-poor aryl substituents can be readily introduced. Also, chloro and fluoro substituents and even unprotected phenol derivatives, which are incompatible with the coupling of acid chlorides, are well tolerated. Although a thienyl substituent can be carried through the sequence, all attempts to react other heteroaryl iodides such as 3- and 4-pyridyl iodides under the standard conditions met with failure.

## 3. Experimental

### 3.1. General

All cross coupling reactions were carried out in oven dried Schlenk or microwave tubes using septa and syringes under a nitrogen or argon atmosphere. Dry tetrahydrofuran and 1,4-dioxane were supplied by a MBraun system MB-SPS-800 solvent purification system. Chemicals were either commercially obtained from ABCR GmbH & Co KG, Acros Organics, Alfa Aesar GmbH & Co KG, Fluka, Merck KGaA, Riedel-de Haën, Sigma-Aldrich Co., and used as supplied or were already available in the research group.

All products were purified via column chromatography on silica gel 60 M (0.04–0.063 mm) from Macherey-Nagel using the flash technique under a pressure of 2 bar. The crude mixtures were absorbed on Celite^®^ 545 (0.02–0.10 mm) from Merck KGaA, Darmstadt before chromatographic purification. The reaction progress was observed qualitatively using TLC Silica gel 60 F_254_ aluminium sheets. The spots were detected with UV light at 254 nm and with aqueous potassium permanganate solution.

^1^H-, ^13^C-, and ^13^C-135 DEPT NMR spectra were recorded on Bruker AVIII-300 spectrometer, using CDCl_3_ or DMSO-d_6_ as solvents. The resonances of CDCl_3_ or DMSO-d_6_ were locked as internal standards (CDCl_3_: ^1^H δ 7.26, ^13^C δ 77.0; DMSO-d_6_: ^1^H δ 2.49, ^13^C δ 39.7). The multiplicities of signals were abbreviated as follows: s: singlet; d: doublet; dd: doublet of doublets; dq: doublet of quartets; ddd: doublet of doublets of doublets; t: triplet; tdd: triplet of doublets of doublets; m: multiplet and br: broad signal. The type of carbon atom was determined on the basis of ^13^C-135 DEPT NMR spectra. For the description of the ^13^C-NMR spectra primary carbon atoms are abbreviated as CH_3_, secondary carbon atoms as CH_2_, tertiary carbon atoms as CH, and quaternary carbon atoms as C_quat_. EI mass spectra were measured on Finnigan MAT 200 spectrometer. IR spectra were either obtained on Shimadzu IRAffinity or on Bruker Vector 22 FT-IR instruments. The intensity of the signals was abbreviated as follows: s (strong), m (medium), w (weak). The melting points (uncorrected) were measured on a Reichert Thermovar apparatus. Combustion analyses were carried out on a Perkin Elmer Series II Analyser 2400 in the microanalytical laboratory of the Institut für Pharmazeutische und Medizinische Chemie der Heinrich-Heine-Universität Düsseldorf.

### 3.2. Experimental Procedure for the Synthesis of 1-(3,3-Diethoxyprop-1-yn-1-yl)-4-methoxybenzene (***3a***)


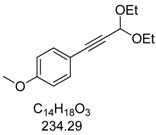


In a screw-cap Schlenk tube PdCl_2_(PPh_3_)_2_ (28 mg, 2 mol%, 0.04 mmol) and CuI (16 mg, 4 mol%, 0.08 mmol) were tempered. Then, 1-iodo-4-methoxybenzene (1a, 278 mg, 2.00 mmol), 3,3-diethoxyprop-1-yne (2, 0.32 mL, 2.20 mmol, 1.1 equiv.), degassed dry 1,4-dioxane (5.00 mL), and triethylamine (0.55 mL, 4.00 mmol, 2.0 equiv.) were successively added under an argon atmosphere. The mixture was stirred for 2 h until complete conversion of **1a** (TLC monitoring). The crude mixture was absorbed onto Celite^® ^and purified chromatographically on silica gel with hexanes/ethylacetate (30:1, *R_f_* = 0.26) to give the alkyne **3a** (415 mg, 1.80 mmol, 89%) as a colorless oil. ^1^H-NMR (CDCl_3_, 300 MHz): δ 1.27 (t, *J* = 7.1 Hz, 6H), 3.65 (dq, *J* = 9.5 Hz, *J* = 7.1 Hz, 2H), 3.80 (s, 3H), 3.81 (dq, *J* = 9.5 Hz, *J* = 7.1 Hz, 2H), 5.48 (s, 1H), 6.80–6.85 (m, 2H), 7.38–7.43 (m, 2H). ^13^C-NMR (CDCl_3_, 75 MHz): δ 15.1 (CH_3_), 55.2 (CH_3_), 60.8 (CH_2_), 83.0 (C_quat_), 85.2 (C_quat_), 91.7 (CH), 113.8 (CH), 113.9 (C_quat_), 133.4 (CH), 159.9 (C_quat_). EI + MS (*m/z* (%)): 234 (M^+^, 7), 205 ((M-C_2_H_5_)^+^, 2), 190 (16), 189 ((M-C_2_H_5_O)^+^, 100), 175 (12), 162 (20), 161 (95), 160 (C_10_H_8_O_2_^+^, 14), 159 (22), 133 (28), 132 (18), 131 (C_9_H_7_O^+^, 10), 118 (12), 89 (15). IR (Oil; *Shimadzu IRAffinity*): 2974 (w) cm^−1^, 2932 (w), 2884 (w), 2839 (w), 2224 (w), 1604 (w), 1570 (w), 1508 (s), 1458 (w), 1443 (w), 1391 (w), 1356 (w), 1329 (w), 1290 (m), 1246 (s), 1172 (m), 1090 (s), 1049 (s), 1032 (s), 1005 (s), 978 (m), 895 (w), 831 (s), 806 (m), 739 (w), 665 (w), 642 (w). Anal. calcd. for: C_14_H_18_O_3_ (234.3): C 71.77, H 7.74; Found: C 71.75, H 7.71.

### 3.3. Typical Experimental Procedure (Synthesis of Pyrazole ***4b***)

In a screw-cap Schlenk tube PdCl_2_(PPh_3_)_2_ (28 mg, 0.04 mmol) and CuI (16 mg, 0.08 mmol) were tempered. Then, 1-iodo-3,4,5-trimethoxybenzene (**1b**, 600 mg, 2.00 mmol), 3,3-diethoxyprop-1-yne (**2**, 0.32 mL, 2.20 mmol), degassed dry 1,4-dioxane (5.00 mL), and triethylamine (0.55 mL, 4.00 mmol) were successively added under an argon atmosphere. The mixture was stirred for 2 h at room temperature (water bath) until complete conversion of **1b** (according to TLC monitoring). Then, hydrazine hydrochloride (280 mg, 4.00 mmol), deionized water (5.00 mL), and PTSA·H_2_O (388 mg, 2.00 mmol) were added. The mixture was stirred at 80 °C (preheated oil bath) until complete conversion (according to TLC monitoring). After extraction with CH_2_Cl_2_ (10 × 10 mL) the combined organic phases were dried with MgSO_4_. The solvents were removed *in vacuo*, the crude mixture was purified by chromatography on silica gel (CH_2_Cl_2_/MeOH/NH_3_ (aq)) to give *3-(3,4,5-tri-methoxyphenyl)-1H-pyrazole* (**4b**, 295 mg, 63%) as an orange solid, Mp 95 °C. ^1^H-NMR (CDCl_3_, 300 MHz): *δ* 3.88 (s, 3H), 3.90 (s, 6H), 6.58 (d, *J* = 1.7 Hz, 1H), 6.99 (s, 2H), 7.61 (d, *J* = 1.8 Hz, 1H). ^13^C-NMR (CDCl_3_, 75 MHz): *δ* 56.1 (CH_3_), 60.9 (CH_3_), 102.5 (CH), 103.0 (CH), 127.9 (C_quat_), 132.6 (CH), 138.0 (C_quat_), 149.5 (C_quat_), 153.5 (C_quat_). EI+MS (*m/z* (%)): 235 (13), 234 (M^+^, 100), 219 ((M−CH_3_)^+^, 68), 205 ((M−HN_2_)^+^, 2), 192 ((M−CH_2_N_2_)^+^, 2), 191 (30), 176 (30), 161 (20), 159 (15), 149 (13), 131 (15), 105 (22), 58 (23), 43 (67). IR (neat; Shimadzu IRAffinity): 3576 (w) cm^−1^, 3146 (w), 3041 (w), 2995 (w), 2963 (w), 2930 (m), 2860 (w), 2826 (w), 1730 (w), 1587 (m), 1516 (m), 1474 (m), 1449 (m), 1416 (m), 1325 (m), 1288 (w), 1261 (w), 1238 (m), 1121 (s), 1092 (m), 1057 (m), 1034 (w), 995 (s), 928 (w), 889 (w), 845 (s), 826 (m), 760 (s), 739 (m), 665 (w), 627 (m). Anal. calcd. for C_12_H_14_N_2_O_3_ (234.3): C 61.53, H 6.03, N 11.96; Found: C 61.63, H 6.29, N 11.68.

The pyrazoles **4a**, **4c–j** were similarly prepared following the general procedure described above. The corresponding reactions conditions and work-ups are summarized in Table 4.

**Table 4:** Synthesized 3-substituted 1*H*-pyrazoles **4** via alkynylation-cyclocondensation sequence.

Entry	(Hetero)Aryl iodide 1	Reaction time *t*	Pyrazole 4	Chromatographic
	(2.00 mmol)	2nd step	(isolated yield)	purification
1	**1a**, 1-Iodo-4-methoxybenzene	15 min	195 mg	CH_2_Cl_2_/MeOH/NH_3_
	*Merck*		(1.12 mmol, 56%)	
	478 mg		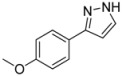	
			**4a**	100:2:1
2	**1b**, 1-Iodo-3,4,5-trimethoxybenzene	15 min	295 mg	CH_2_Cl_2_/MeOH/NH_3_
	*Alfa Aesar*		(1.26 mmol, 63%)	
	600 mg		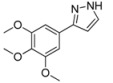	
			**4b**	100:2:1
3	**1c**, 1-Iodo-2-hydroxybenzene	15 min	150 mg	CH_2_Cl_2_/MeOH/NH_3_
	*ABCR*		(0.94 mmol, 47%)	
	449 mg		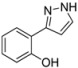	
			**4c**	100:1:1
4	**1d**, 1-Iodo-3-hydroxybenzene	15 min	196 mg	CH_2_Cl_2_/MeOH/NH_3_
	*Alfa Aesar*		(1.23 mmol, 61%)	
	449 mg		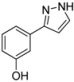	
			**4d**	100:1:1 → 100:3:1 → 100:7:1
5	**1e**, 1-Iodo-4-fluorobenzene	15 min	170 mg	CH_2_Cl_2_/MeOH/NH_3_
	*ABCR*		(1.05 mmol, 53%)	
	0.32 mL		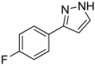	
			**4e**	100:1:1 → 100:2:1
6	**1f**, 1-Iodo-4-chlorobenzene	30 min	121 mg	CH_2_Cl_2_/MeOH/NH_3_
	*ABCR*		(0.68 mmol, 34%)	
	482 mg		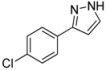	
			**4f**	100:2:1
7	**1g**, 1-Iodo-4-trifluoromethylbenzene	15 min	244 mg	CH_2_Cl_2_/MeOH/NH_3_
	*Alfa Aesar*		(1.15 mmol, 57%)	
	0.30 mL		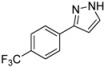	
			**4g**	100:1:1 → 100:2:1
8	**1h**, 1-Iodo-4-cyanobenzene	15 min	90 mg	CH_2_Cl_2_/NH_3_ 100:1 → CH_2_Cl_2_/MeOH/NH_3_
	*ABCR*		(0.53 mmol, 27%)	
	467 mg		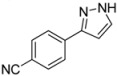	
			**4h**	100:1:1
9	**1i**, 1-Iodo-3-nitrobenzene	15 min	209 mg	CH_2_Cl_2_/MeOH/NH_3_
	*ABCR*		(1.10 mmol, 55%)	
	508 mg		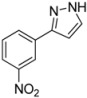	
			**4i**	100:2:1
10	**1j**, 2-Iodothiophene	1 h	151 mg	CH_2_Cl_2_/MeOH/NH_3_
	*ABCR*		(1.00 mmol, 50%)	
	429 mg		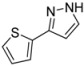	
			**4j**	100:1:1

### 3.4. Spectroscopic Data of 1H-Pyrazoles ***4***

#### 3.4.1. *3-(4-Methoxyphenyl)-1H-pyrazole* (**4a**)


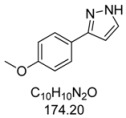


195 mg (1.12 mmol, 56%) as a pale yellow solid. Mp 128 °C. ^1^H-NMR (CDCl_3_, 300 MHz): δ 3.83 (s, 3H), 6.52 (d, *J* = 2.0 Hz, 1H), 6.89–6.94 (m, 2H), 7.59 (d, *J* = 2.1 Hz, 1H), 7.65–7.69 (m, 2H), 10.9 (br, 1H). ^13^C-NMR (CDCl_3_, 75 MHz): δ 55.3 (CH_3_), 102.0 (CH), 114.1 (CH), 124.7 (C_quat_), 127.1 (CH), 133.6 (CH), 148.5 (C_quat_), 159.5 (C_quat_). EI + MS (*m/z* (%)): 175 (12), 174 (M^+^, 100), 159 ((M−CH_3_)^+^, 52), 145 ((M−HN_2_)^+^, 2), 132 ((M−CH_2_N_2_)^+^, 5), 131 (37), 77 (11). IR (Neat; Shimadzu IRAffinity): 3048 (w) cm^−1^, 2965 (w), 2914 (w), 2904 (w), 2824 (w), 2783 (w), 2725 (w), 1611 (w), 1526 (w), 1508 (m), 1454 (m), 1439 (m), 1417 (w), 1275 (m), 1248 (s), 1182 (s), 1113 (w), 1098 (m), 1055 (w), 1026 (s), 952 (m), 934 (m), 897 (m), 853 (m), 831 (s), 795 (m), 773 (s), 729 (w), 611 (m). Anal. calcd. for C_10_H_10_N_2_O (174.2): C 68.95, H 5.79, N 16.08; Found: C 68.73, H 5.48, N 15.88.

#### 3.4.2. *3-(3,4,5-Trimethoxyphenyl)-1H-pyrazole* (**4b**)


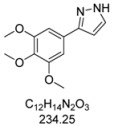


295 mg (1.26 mmol, 63%) as an orange solid. Mp 60 °C. ^1^H-NMR (CDCl_3_, 300 MHz): δ 3.88 (s, 3H), 3.90 (s, 6H), 6.58 (d, *J* = 1.7 Hz, 1H), 6.99 (s, 2H), 7.61 (d, *J* = 1.8 Hz, 1H). ^13^C-NMR (CDCl_3_, 75 MHz): δ 56.1 (CH_3_), 60.9 (CH_3_), 102.5 (CH), 103.0 (CH), 127.9 (C_quat_), 132.6 (CH), 138.0 (C_quat_), 149.5 (C_quat_), 153.5 (C_quat_). EI + MS (*m/z* (%)): 235 (13), 234 (M^+^, 100), 219 ((M-CH_3_)^+^, 68), 205 ((M-HN_2_)^+^, 2), 192 ((M-CH_2_N_2_)^+^, 2), 191 (30), 176 (30), 161 (20), 159 (15), 149 (13), 131 (15), 105 (22), 58 (23), 43 (67). IR (Neat; Shimadzu IRAffinity): 3576 (w) cm^−1^, 3146 (w), 3041 (w), 2995 (w), 2963 (w), 2930 (m), 2860 (w), 2826 (w), 1730 (w), 1587 (m), 1516 (m), 1474 (m), 1449 (m), 1416 (m), 1325 (m), 1288 (w), 1261 (w), 1238 (m), 1121 (s), 1092 (m), 1057 (m), 1034 (w), 995 (s), 928 (w), 889 (w), 845 (s), 826 (m), 760 (s), 739 (m), 665 (w), 627 (m). Anal. calcd. for C_12_H_14_N_2_O_3_ (234.3): C 61.53, H 6.03, N 11.96; Found: C 61.63, H 6.29, N 11.68.

#### 3.4.3. *2-(1H-Pyrazol-3-yl)phenol* (**4c**)


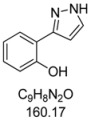


150 mg (0.94 mmol, 47%) as a yellow solid. Mp 92 °C. ^1^H-NMR (CDCl_3_, 300 MHz): δ 6.73 (d, *J* = 2.6 Hz, 1H), 6.93 (ddd, *J* = 7.7 Hz, *J* = 7.3 Hz, *J* = 2.0 Hz, 1H), 7.05 (dd, *J* = 8.2 Hz, *J* = 1.0 Hz, 1H), 7.24 (ddd, *J* = 8.2 Hz, *J* = 7.3 Hz, *J* = 2.0 Hz, 1H), 7.61 (dd, *J* = 7.7 Hz, *J* = 1.6 Hz, 1H), 7.64 (d, *J* = 2.6 Hz, 1H), 9.9–11.2 (br, 2 H). ^13^C-NMR (CDCl_3_, 75 MHz): δ 102.1 (CH), 116.5 (C_quat_), 117.0 (CH), 119.3 (CH), 126.6 (CH), 129.1 (CH), 129.3 (CH), 152.0 (C_quat_), 155.8 (C_quat_). EI + MS (*m/z* (%)): 161 (11), 160 (M^+^, 100), 132 (12), 131 ((M−HN_2_)^+^, 53), 104 (12). IR (KBr; Bruker Vector 22 FT-IR): 3277 (s) cm^−1^, 1736 (w), 1719 (w), 1686 (w), 1655 (w), 1625 (m), 1589 (s), 1560 (w), 1518 (m), 1451 (s), 1399 (m), 1286 (m), 1256 (s), 1200 (m), 1126 (m), 1110 (m), 1080 (m), 1047 (m), 952 (m), 826 (m), 779 (s), 746 (s), 600 (m), 561 (m), 517 (m). Anal. calcd. for C_9_H_8_N_2_O (160.2): C 67.49, H 5.03, N 17.49; Found: C 67.41, H 4.90, N 17.26.

#### 3.4.4. *3-(1H-Pyrazol-3-yl)phenol* (**4d**)


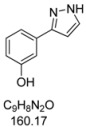


196 mg (1.23 mmol, 61%) as a pale brown solid. Mp 154 °C. ^1^H-NMR (DMSO-d_6_, 300 MHz): δ 6.60 (d, *J* = 2.0 Hz, 1H), 6.68-6.70 (m, 1H), 7.15-7.31 (m, 3H), 7.7 (br, 1H), 9.4 (br, 1H), 12.4–13.5 (2xbr, 1H).^13^C-NMR (DMSO-d_6_, 75 MHz): δ 102.1 (CH), 112.2 (CH), 114.7 (CH), 116.3 (CH), 129.9 (CH), 135.3 (C_quat_), 150.3 (C_quat_), 157.8 (C_quat_). EI + MS (*m/z* (%)): 161 (11), 160 (M^+^, 100), 131 ((M-HN_2_)^+^, 25), 93 (C_6_H_5_O^+^, 3). IR (KBr, Bruker Vector 22 FT-IR): 3245 (s) cm^−1^, 1610 (m), 1591 (s), 1541 (w), 1494 (m), 1478 (s), 1439 (s), 1382 (m), 1292 (m), 1258 (s), 1239 (m), 1218 (m), 1161 (w), 1117 (m), 1097 (m), 1082 (m), 1053 (m), 987 (w), 940 (m), 862 (s), 758 (s), 681 (s), 605 (w), 540 (w). Anal. calcd. for C_9_H_8_N_2_O (160.2): C 67.49, H 5.03, N 17.49; Found: C 67.50, H 5.04, N 17.31.

#### 3.4.5. *3-(4-Fluorophenyl)-1H-pyrazole* (**4e**)


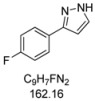


170 mg (1.05 mmol, 53%) as a pale brown solid. Mp 104 °C. ^1^H-NMR (CDCl_3_, 300 MHz): δ 6.56 (m, 1H), 7.07 (t, *J* = 8.7 Hz, 2H), 7.59 (m, 1H), 7.69-7.74 (m, 2H), 11.0-12.2 (br, 1H). ^13^C-NMR (CDCl_3_, 75 MHz): δ 102.5 (CH), 115.7 (d, *J* = 21.7 Hz, CH), 127.5 (d, *J* = 8.1 Hz, CH), 128.6 (C_quat_), 132.5 (CH), 148.9 (C_quat_), 162.6 (d, *J* = 247.1 Hz, C_quat_). EI + MS (*m/z* (%)): 163 (11), 162 (M^+^, 100), 161 (11), 135 (16), 133 ((M-HN_2_)^+^, 27), 108 (11), 95 (C_6_H_4_F^+^, 10). IR (Neat; Shimadzu IRAffinity): 3121 (w) cm^−1^, 3051 (w), 3034 (w), 2959 (w), 2918 (w), 2901 (w), 2882 (w), 2860 (w), 2841 (w), 2814 (w), 2776 (w), 2745 (w), 2725 (w), 2675 (w), 2627 (w), 2581 (w), 1651 (w), 1607 (w), 1524 (m), 1506 (m), 1452 (m), 1408 (w), 1352 (w), 1337 (w), 1233 (s), 1223 (s), 1204 (w), 1157 (m), 1111 (w), 1094 (m), 1082 (w), 1051 (m), 1015 (w), 953 (m), 934 (m), 885 (w), 839 (s), 812 (s), 770 (s), 752 (w), 692 (w), 673 (w), 602 (m). Anal. calcd. for C_9_H_7_FN_2_ (162.2): C 66.66, H 4.35, N 17.27; Found: C 66.92, H 4.41, N 17.00.

#### 3.4.6. *3-(4-Chlorophenyl)-1H-pyrazole* (**4f**)


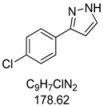


121 mg (0.68 mmol, 34 %) as an orange-brown solid. Mp 95 °C. ^1^H-NMR (CDCl_3_, 300 MHz): δ 6.60 (s, 1H), 7.36 (d, *J* = 8.4 Hz, 2H), 7.60 (s, 1H), 7.69 (d, *J* = 8.5 Hz, 2H), 8.5–12.1 (br, 1H). ^13^C-NMR (CDCl_3_, 75 MHz): δ 102.8 (CH), 127.1 (CH), 128.9 (CH), 131.0 (C_quat_), 132.2 (CH), 133.8 (C_quat_), 149.0 (C_quat_). EI + MS (*m/z* (%)): 180 (M(^37^Cl)^+^, 30), 179 (14), 178 (M(^35^Cl)^+^, 100), 151 ((M−HN_2_)(^37^Cl)^+^, 10), 149 ((M−HN_2_)(^35^Cl)^+^, 4), 116 (10), 115 (19), 113 (C_6_H_4_^37^Cl^+^, 5), 111 (C_6_H_4_^35^Cl^+^, 7), 89 (12). IR (Neat; Shimadzu IRAffinity): 3165 (w) cm^−1^, 2955 (w), 2918 (m), 2876 (w), 2847 (w), 1510 (m), 1447 (m), 1406 (w), 1115 (w), 1090 (s), 1080 (m), 1047 (m), 1013 (m), 953 (m), 924 (m), 878 (w), 833 (s), 761 (s), 723 (m), 692 (m), 613 (m). Anal. calcd. for C_9_H_7_ClN_2_ (178.6): C 60.52, H 3.95, N 15.68; Found: C 60.78, H 4.15, N 15.55.

#### 3.4.7. *3-[4-(Trifluoromethyl)phenyl]-1H-pyrazole* (**4g**)


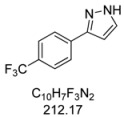


244 mg (1.15 mmol, 57%) as a pale brown solid. Mp 162 °C. ^1^H-NMR (CDCl_3_, 300 MHz): δ 6.68 (m, 1H), 7.62 (m, 1H), 7.63 (d, *J* = 8.1 Hz, 2H), 7.87 (d, *J* = 8.1 Hz, 2H), 10.9–12.7 (br, 1H). ^13^C-NMR (CDCl_3_, 75 MHz): δ 103.0 (CH), 124.1 (q, *J* = 271.9 Hz, C_quat_), 125.7 (q, *J* = 3.8 Hz, CH), 125.9 (CH), 129.8 (q, *J* = 32.5 Hz, C_quat_), 132.0 (CH), 135.9 (C_quat_), 149.1 (C_quat_). EI + MS (m/z (%)): 213 (11), 212 (M^+^, 100), 211 (12), 193 ((M−F)^+^, 7), 185 (11), 183 ((M−HN_2_)^+^, 5), 145 (14). IR (Neat; Shimadzu IRAffinity): 3122 (w) cm^−1^, 3030 (w), 3013 (w), 2959 (w), 2918 (w), 2900 (w), 2868 (w), 2779 (w), 2727 (w), 2679 (w), 1620 (m), 1468 (w), 1416 (w), 1321 (s), 1275 (w), 1182 (s), 1159 (m), 1109 (s), 1097 (s), 1064 (s), 1053 (m), 1013 (m), 957 (m), 930 (w), 881 (w), 841 (s), 783 (m), 770 (s), 741 (m), 692 (m), 673 (w), 617 (w). Anal. calcd. for C_10_H_7_F_3_N_2_ (212.2): C 56.61, H 3.33, N 13.20; Found: C 56.83, H 3.08, N 13.04.

#### 3.4.8. *4-(1H-Pyrazol-3-yl)benzonitrile* (**4h**)


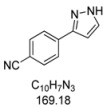


90 mg (0.53 mmol, 27%) as a pale yellow solid. Mp 143 °C. ^1^H-NMR (CDCl_3_, 300 MHz): δ 6.70 (m, 1H), 7.66 (m, 1H), 7.68 (d, *J* = 8.5 Hz, 2H), 7.89 (d, *J* = 8.5 Hz, 2H), 10.4–12.5 (br, 1H). ^13^C-NMR (CDCl_3_, 75 MHz): δ 103.5 (CH), 111.1 (C_quat_), 118.9 (C_quat_), 126.1 (CH), 131.3 (CH), 132.6 (CH), 137.2 (C_quat_), 149.1 (C_quat_). EI + MS (*m/z* (%)): 170 (12), 169 (M^+^, 100), 142 (15), 140 ((M−HN_2_)^+^, 12), 102 (C_7_H_4_N^+^, 7). IR (Neat; Shimadzu IRAffinity): 3248 (w) cm^−1^, 3237 (w), 3129 (w), 3057 (w), 2980 (m), 2972 (m), 2889 (w), 2814 (w), 2220 (w), 1929 (w), 1609 (m), 1508 (w), 1464 (w), 1449 (w), 1393 (w), 1350 (w), 1233 (w), 1221 (w), 1184 (m), 1157 (w), 1074 (w), 1051 (m), 968 (w), 951 (s), 934 (w), 841 (s), 768 (s), 731 (m), 685 (w), 608 (s). Anal. calcd. for C_10_H_7_N_3_ (169.2): C 70.99, H 4.17, N 24.84; Found: C 70.78, H 4.10, N 24.59.

#### 3.4.9. *3-(3-Nitrophenyl)-1H-pyrazole* (**4i**)


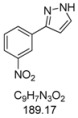


209 mg (1.10 mmol, 55%) as a pale brown solid. Mp 121 °C. ^1^H-NMR (CDCl_3_, 300 MHz): δ 6.74 (d, *J* = 2.3 Hz, 1H), 7.58 (t, *J* = 8.0 Hz, 1H), 7.70 (d, *J* = 2.4 Hz, 1H), 8.15 (tdd, *J* = 8.1 Hz, *J* = 2.2 Hz, *J* = 1.1 Hz, 2H), 8.64 (t, *J* = 1.9 Hz, 1H), 9.8–12.3 (br, 1H). ^13^C-NMR (CDCl_3_, 75 MHz): δ 103.2 (CH), 120.6 (CH), 122.5 (CH), 129.7 (CH), 131.1 (CH), 131.6 (CH), 134.7 (C_quat_), 148.7 (C_quat_), 149.1 (C_quat_). EI + MS (*m/z* (%)): 190 (12), 189 (M^+^, 100), 143 ((M−NO_2_)^+^, 33), 142 (10), 116 (39), 89 (28). IR (Neat; Shimadzu IRAffinity): 3173 (w) cm^−1^, 3067 (w), 2961 (w), 2926 (w), 2851 (w), 1557 (w), 1530 (m), 1518 (m), 1504 (m), 1487 (m), 1398 (w), 1375 (m), 1344 (m), 1315 (w), 1275 (m), 1265 (w), 1221 (w), 1202 (w), 1072 (w), 993 (m), 972 (w), 897 (w), 851 (w), 829 (w), 775 (m), 735 (m), 698 (s), 683 (m), 671 (m), 615 (m). Anal. calcd. for C_9_H_7_N_3_O_2_ (189.2): C 57.14, H 3.73, N 22.21; Found: C 57.19, H 4.02, N 22.18.

#### 3.4.10. *3-(Thiophen-2-yl)-1H-pyrazole* (**4j**)


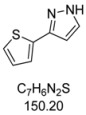


151 mg (1.00 mmol, 50%) as an orange solid. Mp 89 °C. ^1^H-NMR (CDCl_3_, 300 MHz): δ 6.54 (d, *J* = 2.1 Hz, 1H), 7.07 (dd, *J* = 5.1 Hz, *J* = 3.6 Hz, 1H), 7.27 (dd, *J* = 5.1 Hz, *J* = 1.0 Hz, 1H), 7.35 (dd, *J* = 3.6 Hz, *J* = 1.1 Hz, 1H), 7.63 (d, *J* = 2.3 Hz, 1H), 11.55 (s, 1H). ^13^C-NMR (CDCl_3_, 75 MHz): δ 102.7 (CH), 124.2 (CH), 124.6 (CH), 127.6 (CH), 131.5 (CH), 135.7 (C_quat_), 145.6 (C_quat_). EI + MS (*m/z* (%)): 152 (13), 151 (26), 150 (M^+^, 100), 123 (20), 122 (16), 121 ((M−HN_2_)^+^, 35), 105 ((M−CHS)^+^, 7), 96 (22), 78 (12). IR (Neat; Shimadzu IRAffinity): 3146 (w) cm^−1^, 3129 (w), 3117 (w), 3102 (w), 3022 (w), 2957 (w), 2913 (w), 2872 (w), 2855 (w), 2808 (w), 2361 (w), 1732 (w), 1560 (w), 1522 (w), 1464 (w), 1410 (w), 1371 (w), 1273 (w), 1179 (m), 1047 (m), 1030 (m), 910 (m), 845 (m), 824 (m), 756 (s), 723 (w), 692 (s), 644 (w), 602 (m). Anal. calcd. for C_7_H_6_N_2_S (150.2): C 55.97, H 4.03, N 18.65; Found: C 56.12, H 4.12, N 18.46.

## 4. Conclusions

In summary, we have disclosed a novel consecutive three-component synthesis of 3-(hetero)aryl-1*H*-pyrazoles **4** in moderate to good yields starting with the Sonogashira coupling of (hetero)aryl iodides **1** and the commercially available propynal diethylacetal (**2**) as a propargyl aldehyde synthetic equivalent, followed by a rapid sequential acetal cleavage-cyclocondensation with hydrazine hydrochloride. Further studies directed to the synthesis of luminescent pyrazole derivatives as ligands for metal organic frameworks (MOFs) are currently underway.
